# Mechanical Ventilation in Pediatric and Neonatal Patients

**DOI:** 10.3389/fphys.2021.805620

**Published:** 2022-03-17

**Authors:** Michaela Kollisch-Singule, Harry Ramcharran, Joshua Satalin, Sarah Blair, Louis A. Gatto, Penny L. Andrews, Nader M. Habashi, Gary F. Nieman, Adel Bougatef

**Affiliations:** ^1^Department of Surgery, SUNY Upstate Medical University, Syracuse, NY, United States; ^2^Department of Trauma Critical Care Medicine, R Adams Cowley Shock Trauma Center, University of Maryland School of Medicine, Baltimore, MD, United States; ^3^Independent Researcher, San Antonio, TX, United States

**Keywords:** PARDS, lung injury, neonatal and pediatric mechanical ventilation, high frequency percussive oscillation, high frequency oscillatory ventilation, airway pressure release ventilation

## Abstract

Pediatric acute respiratory distress syndrome (PARDS) remains a significant cause of morbidity and mortality, with mortality rates as high as 50% in children with severe PARDS. Despite this, pediatric lung injury and mechanical ventilation has been poorly studied, with the majority of investigations being observational or retrospective and with only a few randomized controlled trials to guide intensivists. The most recent and universally accepted guidelines for pediatric lung injury are based on consensus opinion rather than objective data. Therefore, most neonatal and pediatric mechanical ventilation practices have been arbitrarily adapted from adult protocols, neglecting the differences in lung pathophysiology, response to injury, and co-morbidities among the three groups. Low tidal volume ventilation has been generally accepted for pediatric patients, even in the absence of supporting evidence. No target tidal volume range has consistently been associated with outcomes, and compliance with delivering specific tidal volume ranges has been poor. Similarly, optimal PEEP has not been well-studied, with a general acceptance of higher levels of F_*i*_O_2_ and less aggressive PEEP titration as compared with adults. Other modes of ventilation including airway pressure release ventilation and high frequency ventilation have not been studied in a systematic fashion and there is too little evidence to recommend supporting or refraining from their use. There have been no consistent outcomes among studies in determining optimal modes or methods of setting them. In this review, the studies performed to date on mechanical ventilation strategies in neonatal and pediatric populations will be analyzed. There may not be a single optimal mechanical ventilation approach, where the best method may simply be one that allows for a personalized approach with settings adapted to the individual patient and disease pathophysiology. The challenges and barriers to conducting well-powered and robust multi-institutional studies will also be addressed, as well as reconsidering outcome measures and study design.

## Introduction

Pediatric acute respiratory distress syndrome (PARDS) remains a significant cause of morbidity and mortality, with mortality rates as high as 50% in children with severe PARDS (Schouten et al., 2016). Despite this, pediatric lung injury and mechanical ventilation has been poorly studied, with the majority of investigations being observational or retrospective and with only a few randomized controlled trials (RCTs) (Kneyber et al., 2017). The most recent and universally accepted guidelines for pediatric lung injury are based on consensus opinion rather than objective data (Pediatric Acute Lung Injury Consensus Conference Group, 2015). Therefore, most neonatal and pediatric mechanical ventilation practices have been arbitrarily adapted from adult protocols, neglecting the differences in lung pathophysiology, response to injury, and co-morbidities among the groups.

Neonates, in particular, have a complex set of diseases that require mechanical ventilation but have no adult mimic, such as prematurity, congenital diaphragmatic hernia, meconium aspiration syndrome, persistent pulmonary hypertension, and congenital heart disease (Langham et al., 2003). As a reflection of this unique lung pathophysiology, there are a greater number of pulmonary-related ECMO runs in neonates as compared with adults and pediatric patients (Ecmo, 2021). Babies can be born as early as in the canalicular stages of lung development, where the bronchioles are still not fully developed (Iliodromiti et al., 2013). Applying adult ventilation strategies to neonatal lungs that have yet to form alveoli or cells to produce surfactant is erroneous. Even in children, more similar to adults in terms of their lung disease and co-morbidities, the process of alveolarization is thought to continue even up to 8 years of age (Weibel and Gomez, 1962) and possibly even through adolescence (Narayanan et al., 2012). Additionally, the chest walls of infants are more compliant than the chest walls of adults, with stiffening occurring through the first 2 years of age, at which time the lung and chest wall compliance are nearly equal, as they are in adults (Papastamelos et al., 1995). Thus the pleural pressure and response to mechanical ventilation would be expected to be different in infants (Gattinoni et al., 1998; Kollisch-Singule et al., 2015).

One of the challenges to investigating mechanical ventilation in the pediatric population is that mortality is frequently used as a therapeutic endpoint, but the majority of deaths in pediatric patients on mechanical ventilation are due to neurologic causes rather than refractory hypoxemia (Dowell et al., 2018). Up to 44% of children with no previous respiratory co-morbidities have long-term outcomes of pulmonary dysfunction after a pediatric intensive care unit (ICU) stay for acute respiratory failure including persistent need for adjunct therapies such as oxygen supplementation, bronchodilators, and corticosteroids, or persistent asthma or recurrent pneumonia (Keim et al., 2020). These secondary markers of lung injury may therefore make for a useful alternative marker mortality (Keim et al., 2018).

Further challenges associated with pediatric mechanical ventilation trials includes the lower incidence and mortality as compared with adult patients, shorter duration of mechanical ventilation, and diverse population with an estimated need for 60 participating centers to achieve an adequately powered study (Santschi et al., 2010). This has resulted in a lower number of and less recruited RCTs in children with one of the largest recruited studies having only 153 children (Willson et al., 2005).

Mechanical ventilation modes are grouped similarly as in adults: “conventional” ventilation (CV, including both volume- and pressure- regulated modes), airway pressure release ventilation (APRV), and high frequency ventilation (HFV) which can be subdivided into high frequency oscillatory ventilation (HFOV), high frequency jet ventilation (HFJV), and high frequency percussive ventilation (HFPV). CV is the most commonly used ventilation mode in pediatric patients (Pediatric Acute Lung Injury Consensus Conference Group, 2015; Koopman et al., 2019), with the remaining modes often being used as a rescue for refractory hypoxemia. This is in part because many of the non-CV modes (APRV, HFOV, and HFJV) are able to achieve a higher mean airway pressure without increasing peak airway pressures, and all have unique methods of achieving ventilation. There are few well-powered, recent RCTs in both conventional and non-conventional modes: HFOV (Arnold et al., 1994; Samransamruajkit et al., 2016; Snoek et al., 2016), HFJV (Carlo et al., 1990; Keszler et al., 1991; Keszler, 1997), and APRV (Lalgudi Ganesan et al., 2018). Studies that evaluate non-CV as a rescue mode may be providing an unfair evaluation, as many patients have often been on conventional mechanical ventilation for more than 24 h and have refractory hypoxemia. These studies must therefore be interpreted with caution as they are not necessarily comparing one mode vs. another but whether a non-CV mode can rescue a patient from CV. Although matched cohorts and regression can reduce this bias, they may not fully account for it. There can be no recommendations made for a “best mechanical ventilation” strategy, but it is important to understand the benefits and limitations of the ventilation modes available for clinical use. Thus, the focus of this review is to analyze the studies performed to date on mechanical ventilation strategies in neonatal and pediatric populations.

## Defining Lung Injury

Because of the challenges with conducting well-powered pediatric mechanical ventilation trials, the Pediatric Acute Lung Injury Consensus Conference (PALICC) was held over 2 years with 27 experts voting on 151 recommendations in nine different topics varying from definition of lung injury to treatment and adjunctive therapies (Pediatric Acute Lung Injury Consensus Conference Group, 2015). Although the central premise of developing consensus guidelines is laudable, one must be cautious when using these consensus guidelines and applying them to the individual patient. Of the experts, 22 (81%) were located in the United States and Canada with the remaining five from Spain, Netherlands, England, Switzerland, and Australia, with certain experts hailing from the same institution, suggesting some degree of in-group bias (Pediatric Acute Lung Injury Consensus Conference Group, 2015).

Diagnosing PARDS according to standard criteria (Force et al., 2012) with strict reliance on the P_*a*_O_2_/F_*i*_O_2_ ratio or oxygenation index (OI) is more challenging as children are less likely to have routine arterial blood gases performed. In order to study and compare outcomes in pediatric and neonatal patients, the PALIC Conference determined a definition for PARDS using non-invasive oxygenation criteria. They proposed using an S_*p*_O_2_/F_*i*_O_2_ ratio or oxygen saturation index (OSI) when P_*a*_O_2_ ratio is not available (Pediatric Acute Lung Injury Consensus Conference Group, 2015), as these have been validated in pediatrics (Khemani et al., 2009b). Broadening this inclusion criteria may also help capture children who would otherwise have been excluded due to lack of invasive oxygenation criteria from blood gases (Santschi et al., 2010), also leading to an under-recognition of lung injury (Khemani and Newth, 2010). Embracing non-invasive oxygenation criteria can increase patient eligibility by an estimated 25% (Khemani et al., 2009b). Using this more inclusive definition, the follow-up observational PARDIE study revealed that the PALICC definition of severe PARDS was associated with higher mortality (32.7%) but mortality rates were similar between mild (12.4%) and moderate (10.3%) PARDS (Khemani et al., 2019).

**FIGURE 1:**
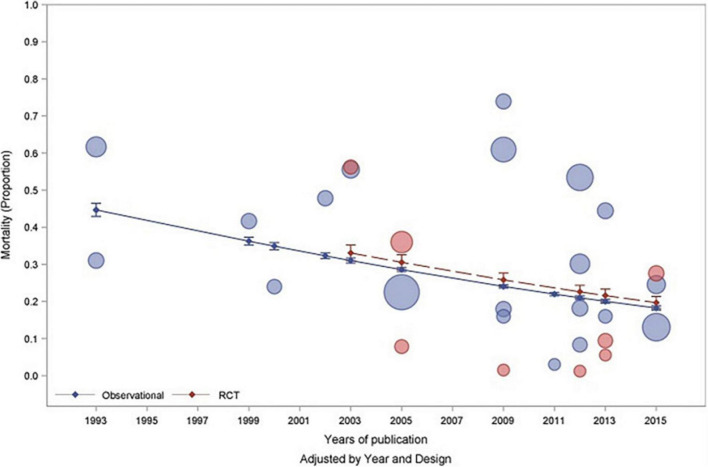
Published with permission from Wong et al. (2019) Bubble plot demonstrating mortality rates associated with pediatric acute respiratory distress syndrome by year of study and study design (observational—blue and RCT—red). The size of the bubbles are proportional to the total number of patients recruited into the individual study.

In a meta-analysis of observational and randomized controlled trials investigating PARDS, the overall pooled mortality was determined to be 24% with a marked reduction in mortality over two decades from 40% mortality to an 18% mortality (Figure 1; Wong et al., 2019). This improvement in survival is likely due to an increased recognition of milder forms of PARDS, improvement in mechanical ventilators and strategies, and also suggests that children overall fare better than adults, likely due to a decrease in co-morbidities. It is important to consider the results of this meta-analysis (Wong et al., 2019) when comparing studies because trials spanning several years may not accurately reflect differences between ventilator modes but rather with patient inclusion, and advancement in ventilator practices and adjunctive therapies.

## Conventional Mechanical Ventilation

**FIGURE 2:**
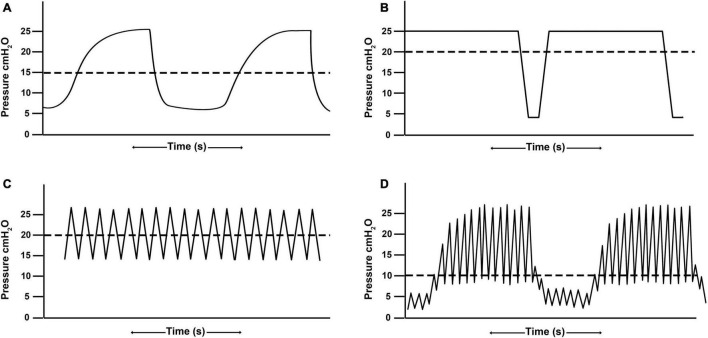
Demonstrative waveforms of **(A)** conventional ventilation **(B)** airway pressure release ventilation, **(C)** high frequency oscillatory ventilation, and **(D)** high-frequency percussive ventilation. Airway pressure release ventilation and high frequency oscillatory ventilation maintain alveolar recruitment by achieving a higher mean airway pressure (horizontal dashed line) as compared with conventional ventilation without raising peak inspiratory pressures.

Conventional ventilation is generally the application of a tidal volume or set inspiratory pressure to a baseline positive-end expiratory pressure (PEEP) with an inspiratory time set shorter than the expiratory time (Figure 2A). The majority of pediatric patients (75.2%) are placed on CV with 26.6% of those patients ventilated with a volume control mode and the remainder ventilated with a pressure control or regulated mode (Santschi et al., 2010). There is marked variability in the management of pediatric patients placed on conventional ventilation (Santschi et al., 2010), suggesting the term “conventional” may be a misnomer as there is not yet a convention in terms of mode (pressure- vs. volume-), tidal volume, or PEEP strategy.

Low tidal volume ventilation has become standard-of-care after the ARDSnet trial in adults demonstrated that 6 mL/kg significantly reduced mortality as compared with 12 mL/kg (ARDSnet, 2000). For lack of a similar comparison trial, low tidal volume ventilation has been mostly adopted into the mechanical ventilation strategy of pediatric patients (Pediatric Acute Lung Injury Consensus Conference Group, 2015; Koopman et al., 2019). In neonates, there are similarly no trials supporting one tidal volume goal over another, but tidal volume targets of 5–6 mL/kg are generally well-accepted (Keszler et al., 2009; Patel et al., 2009).

Although translating the practice of low tidal volume ventilation to pediatric patients has been advocated (Hanson and Flori, 2006; Dellinger et al., 2008), and is generally the standard to which other ventilator modes are compared, there are no large well-powered studies on it (Santschi et al., 2010; Koopman et al., 2019). One observational study demonstrated that higher tidal volumes were affiliated with decreased mortality (Erickson et al., 2007) and neonates were less susceptible to lung injury and inflammation with supraphysiologic tidal volumes (Copland et al., 2004; Kornecki et al., 2005; Smith et al., 2010). In a meta-analysis of eight randomized clinical trials and observational studies, there was no association between tidal volumes (7, 8, 10, 12 mL/kg) and mortality both in patients with and without ARDS (de Jager et al., 2014).

Tidal volumes in children tend to average around 8 mL/kg [8.0 mL/kg (Erickson et al., 2007), 8.1 mL/kg (Albuali et al., 2007), and 8.3 mL/kg (Santschi et al., 2010) but with marked variability (with a range from < 4 to > 15 mL/kg], highlighting the absence of a consensus (Santschi et al., 2010). Even PEEP guidelines have not been standardized (Villar, 2005) with more than half of pediatric patients being placed on PEEP < 5 cm H_2_O with a range of 0–15 cm H_2_O (Santschi et al., 2010). Pediatric intensivists are also more likely to adopt a low PEEP-high F_*i*_O_2_ strategy as compared with adults (Khemani et al., 2009a; Koopman et al., 2019). This is particularly relevant because exposure to high F_*i*_O_2_ concentrations leads to increased alveolar surface tension, in part due to increased surfactant frangibility (Smallwood, 2017). In addition to an unintended consequence of decreased pulmonary compliance and potential exacerbation of an underlying lung injury, higher F_*i*_O_2_ concentrations have been associated with bronchopulmonary dysplasia and longer supplemental oxygen requirements in neonates (No Authors List, 2000; Askie et al., 2003). Caution is deserved when comparing another ventilator mode with “conventional” ventilation since the comparisons will be between one (and possibly two) modes that do not have standardized methods of setting ventilator parameters and a wide variability in practice.

## Airway Pressure Release Ventilation

Airway Pressure Release Ventilation (APRV) is a pressure-regulated, time-dependent mode where the upper pressure (P_High_) is sustained for a prolonged time (T_High_), creating a continuous positive airway pressure (CPAP) phase to allow for alveolar stabilization and recruitment. The CPAP Phase is interrupted with a lower pressure (P_Low_) for a brief [millisecond] period of time (T_Low_) creating a Release Phase to allow for ventilation (Figure 2B; Habashi, 2005). A primary advantage to APRV is that it can achieve higher mean airway pressure, promoting recruitment and oxygenation, while limiting peak inspiratory pressures (Anderson and Speicher, 2006). It also allows for spontaneous breathing (Krishnan and Morrison, 2007), thereby increasing patient comfort and limiting need for neuromuscular blockade (Frawley and Habashi, 2004; Krishnan and Morrison, 2007). Spontaneous breathing additionally allows for distribution of gas to the dependent regions of the lung, promoting further alveolar recruitment (Habashi, 2005). Although there is a wide variability in APRV settings, as with any mode (Jain et al., 2016), the most frequently cited is the Time-Controlled Adaptive Ventilation (TCAV™) method which precisely sets the time at expiration (T_Low_) based on the expiratory flow curve to optimize ventilation while limiting alveolar derecruitment (Frawley and Habashi, 2004; Habashi, 2005; Jain et al., 2016; Nieman et al., 2020).

Although APRV has been an available ventilator mode since 1987 (Stock et al., 1987), it is not widely used in pediatric or neonatal populations (Krishnan and Morrison, 2007). APRV is largely considered a rescue ventilator mode (Gupta et al., 2013) and the incidence of use in pediatric patients in the literature is just 1.6% as compared with 11.3% in adults (Gupta et al., 2013). This lack of clinical experience suggests that patient management can be quite variable from one intensivist to another (Anderson and Speicher, 2006). The sparse APRV studies must therefore be interpreted with caution with close attention to the method by which the mode was set.

## Pediatric Airway Pressure Release Ventilation

In pediatric patients, APRV is set similar to adult patients with a P_High_ set at the plateau pressure achieved in the CV mode or at the mean airway pressure on HFOV plus 2–4 cm H_2_O (Habashi, 2005). Pediatric patients naturally have a higher respiratory rate and minute ventilation as compared with adults and therefore the T_High_ is often set shorter. As compared with adults with healthy lungs (where the T_High_ is set at 4–6 s), pediatric patients with healthy lungs will have the T_High_ set at 3–5 s. In pediatric patients with injured, derecruited lungs, the T_High_ may be set at a shorter duration of 1–3 s to allow for bulk ventilation. Generally, the longest possible T_High_ is selected that maintains adequate CO_2_ clearance (Frawley and Habashi, 2004; Habashi, 2005). The T_Low_ is adjusted as in adults to terminate the expiratory flow at 75% of the peak expiratory flow (Habashi, 2005).

In a prospective, randomized, crossover clinical trial of 15 pediatric patients with mild to moderate lung disease, APRV was found to have similar oxygenation, ventilation, hemodynamics, and patient comfort as compared with SIMV but with a lower peak inspiratory and plateau pressures. The shortcoming of this study was the absence of reported ventilator settings for either group (Schultz et al., 2001). One of the cited concerns for APRV is whether there are hemodynamic consequences given the higher mean airway pressure. In a small case series of pediatric patients, transition to APRV was associated with no alteration of hemodynamics and no need for neuromuscular blockade, while improving oxygenation (Krishnan and Morrison, 2007). Similarly, in patients with refractory hypoxemia transitioned from CV, APRV reduced neuromuscular blockade requirements as compared with HFOV, however, the HFOV group was represented by a younger cohort with a higher OI (Yehya et al., 2014a).

The only published RCT applying APRV to pediatric patients was performed by Lalgudi Ganesan et al. (2018) This was a single-center study conducted over a 2.5-year period but was terminated after 50% enrollment (52 children) due to higher mortality in the APRV arm. This study must be interpreted thoughtfully because it does not necessarily suggest that the APRV mode is harmful but rather that the method of setting it may have been. The authors adjusted the P_High_ to maintain a release tidal volume of 6–7 mL/kg ideal body weight (Lalgudi Ganesan et al., 2018). In doing so, the patients were placed on an open lung technique designed to recruit the lung and number of alveoli available to accommodate a larger tidal volume, but restricted the tidal volumes (Frawley and Habashi, 2004). By comparison, the TCAV^TM^ method does not restrict tidal volumes as the lung opens. Rather, larger tidal volumes are viewed as evidence of increasing lung recruitment and improved compliance (Kollisch-Singule et al., 2014).

## Neonatal Airway Pressure Release Ventilation

In neonatal patients transitioning to APRV, the P_High_ is similarly set at the plateau pressure achieved in the CV mode or at the mean airway pressure on HFOV plus 0–2 cm H_2_O. Neonates have a further decrease in their set T_High_ to 1–2 s with the T_Low_ adjusted to terminate the expiratory flow at 75% of the peak expiratory flow (Habashi, 2005). No large studies of APRV have been performed in neonates to date. Gupta et al. (2013) reported a case series of 5 infants ranging in gestational age from 24 to 28 weeks and found that the infants tolerated APRV well with no adverse events.

In a neonatal lamb model with oleic acid induced lung injury, APRV demonstrated improved oxygenation and ventilation as compared with CPAP. APRV and CV had similar ventilation and oxygenation but APRV achieved this with a lower peak airway pressure and with no hemodynamic instability (Martin et al., 1991). In a 24-h model of respiratory distress syndrome, piglets were birthed at the equivalent of a 28-week human gestation. APRV set and adjusted by the TCAV™ method led to increased lung recruitment, improved ventilation, and decreased oxygen requirements without altering hemodynamics (Arrindell et al., 2015). In a subsequent 48-h porcine model of respiratory distress syndrome where piglets were birthed at the equivalent of a 25-week human gestation, APRV set according to the TCAV™ method led to a significant increase in lung compliance with a trend toward improved oxygenation with lower oxygen requirements (Kollisch-Singule et al., 2017).

## High-Frequency Oscillatory Ventilation

High frequency oscillatory ventilation operates using a push-pull application of pressure to the airway opening by either piston/diaphragm or microprocessor gas controllers. Fresh gas is supplied within the ventilator circuit as a bias flow, and mean airway pressure is adjusted according to the relationship between fresh gas inflow and any positive or negative pressure placed on the gas outflow from the bias flow circuit. The clinician has the ability to set the oscillatory frequency, pressure amplitude (Δ), oscillator displacement (volume), inspiratory/expiratory ratio, and bias flow. The mean airway pressure or continuous distending pressure (CDP) is generally set at higher value to improve alveolar recruitment and oxygenation (Figure 2C). Carbon dioxide elimination is correlated with the coefficient of gas transport (DCO_2_), which is the product of frequency (f) and tidal volume-squared (f * Vt^2^). Thus, a high DCO_2_ leads to an improvement in ventilation, as modified by frequency and especially the tidal volume. Increases in frequency can, however, lead to decreases in DCO_2_ by secondarily decreasing tidal volume unless the pressure amplitude is simultaneously increased (Sanchez Luna et al., 2013).

High frequency oscillatory ventilation is the most commonly used HFV mode with 16.4% of patients receiving HFOV in a cross-sectional observational study, but with the majority of patients placed on CV (Santschi et al., 2010). This is higher than the 2.9% reported by Arnold et al. (2000) 10 years earlier, which could reflect institutional bias or an increased acceptance of the mode. Intensivists are more apt to start HFOV on infants as an early therapy as compared with their pediatric counterparts, but it is still generally considered a rescue therapy in pediatric populations (Ben Jaballah et al., 2006). Patients are on CV between 2.2 to 11.4 days before being switched to HFOV and with an OI ranging from 27.1 to 36.7 (Arnold et al., 2000). HFOV is also the most studied of the HFV modes, but indications, timing, and strategy of HFOV remain poorly defined (Kneyber et al., 2012). Also, there was a significant difference in performance among earlier generation high-frequency oscillators such that similar settings may have generated opposing results, especially in terms of gas exchange, barotrauma, and intraventricular hemorrhage/periventricular leukomalacia (Jouvet et al., 1997). This is likely contributing to disparate results across earlier studies, particularly when applied to patients who have been transitioned from CV due to refractory hypoxemia. Technological advances have markedly improved the high-frequency oscillators that are available for clinical use such that individual ventilator differences may be less critical. However, even newer generation oscillators have substantive differences ranging from volume delivery and frequency range to required ancillary equipment and available features (Pillow, 2015). Performance among high-frequency oscillators therefore also varies, with discrepancies identified between the set and delivered ΔP in certain machines and disparate tidal volume delivery generation, especially at higher frequencies (Pillow et al., 2001; Tingay et al., 2015). It is therefore important for clinicians to recognize these nuances and understand the high-frequency oscillator that is being applied to the patient in order to improve performance (Pillow, 2015).

## Pediatric High Frequency Oscillatory Ventilation

In a RCT of 70 patients, Arnold et al. (1994) demonstrated that HFOV led to an improvement in oxygenation with an increased mean airway pressure but decreased peak airway pressures, however, the study was underpowered to detect significant differences (Arnold et al., 1994). In that study, up to 66% of patients in the CV group crossed over to the HFOV group but only 38% of patients in the HFOV group crossed over to the CV group with the patients not analyzed in the initially randomized group (Arnold et al., 1994). In a smaller RCT, HFOV combined with recruitment maneuvers led to superior oxygenation as compared with CV without a marked change in hemodynamics (Samransamruajkit et al., 2016).

HFOV has been associated with both a shorter (Wong et al., 2020) and longer (Gupta et al., 2014; Curley et al., 2015) intensive care unit length of stay, which may partly be explained by the timing of HFOV application and disease severity. One retrospective study found a shorter duration ICU stay but increased mortality, suggesting that HFOV does not improve outcomes in patients with fatal lung injury, but patients with recoverable lung disease may benefit from improved oxygenation and a decrease in lung injury (Yehya et al., 2014a,b; Wong et al., 2020). In a larger retrospective review of over 9,000 patients from 98 hospitals, the use of HFOV was associated with longer duration of ventilation and ICU length of stay, as well as a higher mortality (Gupta et al., 2014). These results might be explained by the study design in which patients were matched according to propensity score matching rather than pulmonary disease type or ventilator parameters (Gupta et al., 2014). In the patients who were placed on HFOV and survived, earlier application of HFOV (within 24 h) was associated with a shorter ventilation course and length of stay as compared with patients in whom HFOV was applied later (Gupta et al., 2014).

In contrast, the results of a secondary analysis of the Randomized Evaluation of Sedation Titration for Respiratory Failure (RESTORE) trial (Curley et al., 2015) found that earlier application of HFOV was correlated with a greater length of mechanical ventilation as compared with later application of HFOV and conventional mechanical ventilation, however, there was no association with mortality (Bateman et al., 2016). The combination of these results is confusing and the discrepancies may suggest that different methods of setting HFOV were used among patients and between studies. It is important to recognize that the HFOV settings that are applied initially or early will be different from those applied later. The goal for both is to achieve a homogeneously aerated lung, but the acutely injured lung must be nudged open slowly, particularly if it has been subject to a prolonged time on non-open-lung strategies (Froese and Kinsella, 2005).

Optimal settings of HFOV have not been thoroughly illuminated (Kneyber et al., 2012). Most protocols do not involve recruitment maneuvers (Kneyber et al., 2012) and set frequency somewhat arbitrarily based on patient age and weight in the range of 5–8 Hz (Froese and Kinsella, 2005; Kneyber et al., 2012; de Jager et al., 2019), where frequency should rather be optimized to minimize the pressure cost of ventilation to reduce lung injury (Venegas and Fredberg, 1994). As an example of the challenges of standardizing HFOV, the power or amplitude is adjusted according to the chest wiggle factor, or the level to which the chest wiggles on HFOV (Meyers et al., 2019). This is hardly precise and difficult to standardize across intensivists, and especially so among institutions. Animal models have been designed to assist in determining optimal means of setting HFOV. In a saline lavage injury in lambs, stepwise escalation in mean airway pressure modified lung volume and optimized lung recruitment (Pellicano et al., 2009). The ideal method of setting HFOV would be with a mean airway pressure sufficient to stabilize alveoli but as low as reasonable possible, with the smallest superimposed oscillations to minimize alveolar strain (Kneyber and Markhorst, 2016).

## Neonatal High Frequency Oscillatory Ventilation

One of the earliest randomized studies in 1989 comparing CV vs. HFOV was the HiFi study (Group, 1989) in preterm infants (750–2,000 g), in which HFOV did not reduce mortality or BPD rates (Group, 1989; No Authors List, 1990a). They found an increased rate of intraventricular hemorrhage (IVH) and periventricular leukomalacia (PVL) (Group, 1989). In a follow-up study at 16–24 months post-term age, respiratory status was similar between the two groups but neurodevelopmental outcome were worse in the HFOV group with a correlation between cognitive defects and hydrocephalus with IVH (No Authors List, 1990b).

Although HFOV is often used as a rescue therapy with success, it has not yet become a primary mode to use early on ventilated patients. To study this, in 1996, the multicenter Provo trial reported that early application of HFOV to premature newborns born less than 35 weeks gestation with moderate to severe respiratory distress resulted in decreased lung injury, improved oxygenation, and even a lower incidence of necrotizing enterocolitis (Gerstmann et al., 1996). A follow-up study published 5 years later determined that there was no difference in childhood neurodevelopmental outcomes, however, the CV group had some markers of obstructive lung pathology with a decrease in peak expiratory flow but an increase in residual volume (Gerstmann et al., 2001). This study was especially important to illustrate that reducing pulmonary morbidity in neonates can decrease subsequent pulmonary dysfunction into childhood (Gerstmann et al., 2001).

To summarize, in three longer-term observational studies evaluating the neurodevelopmental outcomes of preterm infants with respiratory distress syndrome, one demonstrated worse neurodevelopmental outcome in the HFOV group (No Authors List, 1990b), one showed similar neurodevelopmental outcome in the HFOV group but improved respiratory function (Gerstmann et al., 2001), and another demonstrated similar neurodevelopmental and respiratory outcomes (Marlow et al., 2006). These changes can be attributed to improvement in adjunctive strategies, comfort level with HFOV, and improvement in methods of setting HFOV. For instance, surfactant therapy was not a routine treatment during the HiFi study (Courtney et al., 2002). There was also a higher cross-over rate from HFOV to conventional ventilation which could be interpreted as less comfort with the ventilator given that the methods reported that the oscillator was selected after bench testing available machines (Group, 1989). The oscillator chosen (Senko Medical Instrument Manufacturing, Tokyo, Japan) only has the ability to deliver an I:E ratio of 1:1 leading to higher CDP, and contributing to a higher incidence of barotrauma, and IVH (Bryan and Froese, 1991). The HiFi study also used a low volume HFOV strategy, which is now thought to be sub-optimal for infants (Bryan and Froese, 1991). Nevertheless, with conflicting results from these three RCTs, it is not surprising that there is a lack of consensus on ventilating neonatal patients.

In a later multicenter clinical trial, Courtney et al. (2002) randomized very low birth weight infants (601–1,200 g) to HFOV or synchronized intermittent mandatory ventilation and revealed that infants were more likely to be extubated early with HFOV and with a decreased rate of supplemental oxygen requirements by 36 weeks postmenstrual age. This study did not reveal a difference in IVH or PVL (Courtney et al., 2002). In a separate randomized comparison of pre-term infants born < 30 weeks of age, HFOV led to decreased surfactant requirements but no improvement in pulmonary outcomes (Moriette et al., 2001).

Other studies have revealed that HFOV is successful in preventing ECMO (Erdeve et al., 2019) and with similar (Snoek et al., 2016) or decreased (Erdeve et al., 2019) mortality rates. It has been affiliated with a lower (Courtney et al., 2002) and similar (Snoek et al., 2016) incidence of chronic lung disease and similar rates of death and IVH (Clark et al., 1994). A Cochrane review of elective HFOV RCTs spanning as early as the HiFi trial determined that the 28-and 30-day mortality between HFOV and CV was similar. They also found an increase in pulmonary air leaks in the HFOV group, a decrease in severe retinopathy of prematurity, and a decrease, albeit inconsistent, in chronic lung disease (Cools et al., 2015).

To further illustrate the importance of fully understanding studies, a meta-analysis of 17 randomized trials comparing HFV to CV determined that changes in outcomes between HFV and CV are more likely due to the method by which the mode was set as compared with the mode itself (Thome et al., 2005). Adjunctive therapies in pediatric and neonatal ICU patients have been rapidly progressing, including surfactant therapy and nitric oxide application. Studies must therefore also be taken in context with the year they were performed (Courtney et al., 2002). Recognizing that with the arsenal of critical care techniques available, the limits of what can be done has also expanded, where studies were previously reporting infants born < 35 weeks of age and weighing < 1.751 kg in 1992 (Clark et al., 1994) to infants weighing as little as 601 g in 2002 (Courtney et al., 2002).

## High-Frequency Percussive Ventilation

**FIGURE 3:**
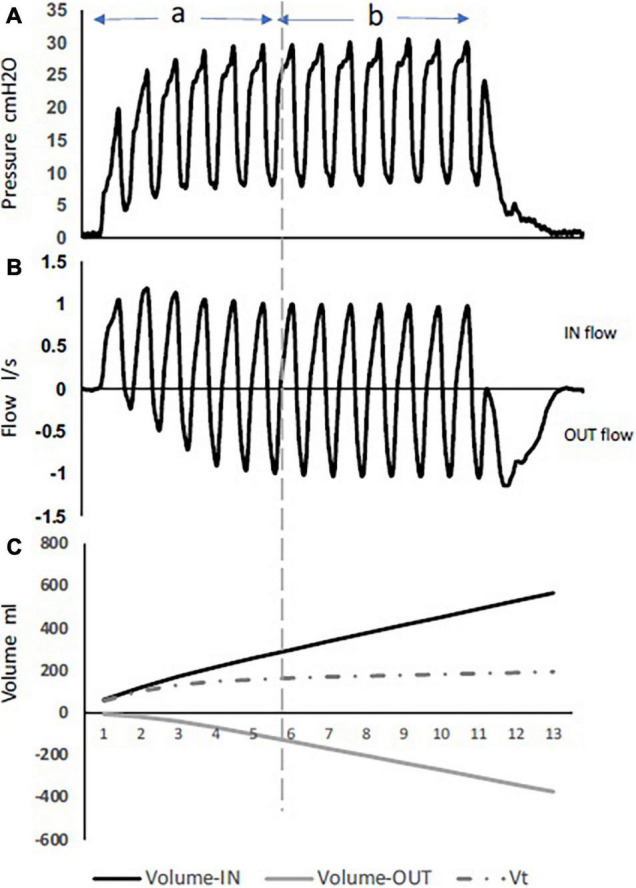
Pressure, flow, and volumes during HFPV inspiratory interval, using a lung model. During phase **(A)** full flow acceleration with an increase in volume-in delivery, while phase **(B)** is characterized with more volume-out. **(C)** The difference between volume-in and volume-out is the tidal volume. Note the tidal volume value remains stable during the whole plateau equilibrium.

High-frequency percussive ventilation (HFPV) is delivered by a pneumatically powered, flow-regulated, time-cycled, pressure-controlled ventilator. HFPV delivers a small tidal volume (or sub-tidal volume) at a high frequency, in combination with a low frequency bulk distribution of gas similar to that of a pressure limited CV breath (Figure 2D). The most unique feature of HFPV is the breathing circuit and the patient airway interface. It uses a sliding venturi, operating simultaneously as inhalation and exhalation valves. It is permanently open to ambient, through which sub-tidal breaths are delivered into the lungs. By venturi effect, flow volume delivery is always inversely proportional to the pressure reached at the level of the airway. The high frequency flow interrupter generator of the ventilator allows the sliding venturi to be regulated in such a manner that there is a stepwise increase in airway pressure during inspiration to the scheduled peak inspiratory pressure (Figure 3; Bougatef et al., 2007).

Two phases describe the inspiration period during HFPV. The first is an initial phase characterized by an inspiratory flow acceleration with a progressive increase in airway pressure. As the inspiratory interval progresses, an expiratory flow component develops. The second phase is where the pressure and flow reach an oscillating plateau equilibrium at the scheduled peak inspiratory pressure, and where the flow signal is characterized by an equilibrium between the inspiratory flow and the expiratory flow components (Figure 3). While the second phase is user-controlled (pressure, amplitude, time), the initial phase is dependent on the patient’s thoraco-pulmonary mechanics. Unlike the other high-frequency modes (HFOV, HFJV), HFPV does not rely on establishing a higher mean airway pressure to maintain alveolar recruitment. Instead, HFPV maintains gas distribution by synergizing with the time constants of the lung compartments, maintaining open alveoli while preventing overinflation (Lucangelo et al., 2010).

During inspiration, the flow is interrupted between two consecutive pulses, resulting in a pressure drop to a value that depends on the thoraco-pulmonary mechanics. The gradual stacking of the successive pulsed volumes results in a progressive increase in lung volume. Like flow and pressure, sub-tidal volume deliveries during the inspiratory interval follows two phases. At the beginning of inspiration, pulsed volume-in are larger in size, while the exhaled volumes (volume-out) are small. As the inspiratory interval progress, pulsed volume-in decreases and volume-out increases in size to reach an equilibrium characterizing the second phase (Figure 3).

After each volume-in, the pulsed flow is interrupted, allowing the exhalation port to vent the proximal airway to ambient, and a volume-out is exhaled by the patient. This represents an important beneficial mechanism of HFPV in the improvement of gas exchange. The tidal volume is calculated by the difference between the cumulative volume-in and the cumulative volume-out (Figure 3).

## Pediatric High-Frequency Percussive Ventilation

In a retrospective observational study of 31 patients who failed conventional ventilation, application of HFPV led to improved oxygenation and ventilation while decreasing the peak inspiratory pressure from 38 to 26 cm H_2_O (Rizkalla et al., 2014). HFPV has found particular application to patients with acute burn injury and respiratory failure, likely because it has been effective in clearing secretions due to percussive bursts (Allan et al., 2010). Three RCTs (Carman et al., 2002; Mlcak et al., 2002; Reper et al., 2002) and one case-controlled series (Cortiella et al., 1999) cumulatively demonstrated improved oxygenation (Cortiella et al., 1999; Carman et al., 2002; Reper et al., 2002) a decrease in peak inspiratory pressures (Cortiella et al., 1999; Carman et al., 2002), and decreased rates of pneumonia (Cortiella et al., 1999; Mlcak et al., 2002) in mechanically ventilated pediatric patients with burns and/or inhalation injury ventilated with HFPV as compared with CV.

## Neonatal High-Frequency Percussive Ventilation

High-frequency percussive ventilation is less well-reported in neonatal studies, often being lumped together with other HFV modes. Only small case series exist in neonates ventilated with HFPV (Pfenninger and Gerber, 1987; Pfenninger and Minder, 1988; Paviotti et al., 2014), but have demonstrated improvements in oxygenation without increasing airway pressures (Pfenninger and Gerber, 1987; Paviotti et al., 2014), and improved static compliance (Pfenninger and Minder, 1988). Nasal high-frequency percussive ventilation has recently been popularized (De La Roque et al., 2011; Moresco et al., 2020; Renesme et al., 2020) and compared with nasal continuous positive airway pressure, demonstrated non-inferiority (Renesme et al., 2013), possible benefit, and that it is well-tolerated (De La Roque et al., 2011). In a piglet model of meconium aspiration syndrome, HFPV and CV were associated with lower mean airway pressures and OI as compared with HFOV, but there was no apparent difference in histologic lung injury (Renesme et al., 2013).

### High-Frequency Jet Ventilation

High frequency jet ventilation is seldom used in adults and most often reported in neonates, particularly in preterm infants (Miller et al., 2021a). With HFJV, a high velocity gas jet is delivered *via* an adapter or jet injector inserted into the endotracheal tube. Similar to HFOV, it delivers small tidal volumes at a rapid rate of up to 660 cycles/min with an inspiratory time set as short as 0.02 s (Smith et al., 1993; Miller et al., 2021a). Unlike HFOV, HFJV is set on top of a conventional ventilation mode which regulates the mean airway pressure (Miller et al., 2021a). Additionally, exhalation is passive and dependent on the rate and inspiratory to expiratory ratio, but allows for marked CO_2_ elimination (Miller et al., 2021a). The jet pulses and continuous air stream are also hypothesized to improve mucous clearance (Miller et al., 2021a). Like the other HFV modes, HFJV is largely used as a rescue mode when CV has failed (Smith et al., 1993).

## Pediatric High Frequency Jet Ventilation

No RCTs exist in the pediatric population and the majority of studies are case series (Miller et al., 2021a), but it has been deemed safe for use as evident in a case series of eleven pediatric patients with respiratory syncytial virus (Valentine et al., 2016). In a physiologic comparison of HFJV vs. CV, HFJV was found to generate a higher intrinsic PEEP leading to an increase in end-expiratory lung volume and improved oxygenation, while also achieving a higher minute ventilation (Berner et al., 1993). In support of this, a retrospective review of 35 pediatric critical care patients (including neonates) transitioned from CV to HFJV were found to have a decrease in acidosis and oxygen requirements (Miller et al., 2021b). HFJV has also been shown to resolve clinically relevant pulmonary barotrauma and air leaks caused by CV at lower mean airway pressures (Smith et al., 1993).

## Neonatal High Frequency Jet Ventilation

In two neonatal RCTs, HFJV was found to improve ventilation at lower peak airway pressures (Keszler et al., 1991; Keszler, 1997). Cross-over was allowed in one of the trials, and 84% of patients who crossed over from CV to HFJV responded well to HFJV, whereas only 9% of those who crossed over from HFJV to CV were successful (Keszler et al., 1991). The incidence of chronic lung disease was similar between CV and HFJV in that study (Keszler et al., 1991), whereas in the other RCT of preterm infants <36 weeks, HFJV was found to reduce the incidence of BPD as well as the need for future supplemental oxygenation (Keszler, 1997). Two different methods of setting HFJV were used in that study: a low airway pressure (Low P_*aw*_) strategy and an optimal volume (OV) strategy. In a subgroup analysis, the HFJV-OV strategy was found to have an improvement in oxygenation whereas the HFJV-Low P_*aw*_ had an increase in ventilation (Keszler, 1997). Other observational studies comparing HFJV with CV have revealed similar oxygenation, ventilation, and hospital days (Wiswell et al., 1996) and no difference in mortality, bronchopulmonary dysplasia, or cross-overs (Carlo et al., 1990), but similar rates of air leaks has been consistent across studies (Carlo et al., 1990; Keszler et al., 1991; Keszler, 1997).

Neurologic outcomes with HFJV have been conflicting. In one of the RCTs, the HFJV-OV strategy was affiliated with a decreased incidence in IVH and PVL as compared with the CV and HFJV-Low P_*aw*_ groups (Keszler, 1997), whereas the other found no difference (Keszler et al., 1991). In another study of 73 premature infants born less than 33 weeks, HFJV had a higher incidence of PVL or poor neurologic outcome (Wiswell et al., 1996). In another study of 42 infants with severe respiratory distress syndrome there was no difference in IVH incidence between HFJV and CV (Carlo et al., 1990).

Other special considerations that have been studied with HFJV include congenital heart disease, congenital diaphragmatic hernias, and persistent pulmonary hypertension. HFJV in infants with congenital heart disease were found to have decreased acidosis and improved ventilation but no significant difference in oxygenation (Miller et al., 2021c). HFJV has demonstrated improved ventilation in infants with congenital diaphragmatic hernias (Zhang et al., 2013) and a trend toward improved survival as compared with CV (Kuluz et al., 2010). HFJV has been compared against HFOV in infants with persistent pulmonary hypertension (Coates, 2008), but no randomized controlled trials exist (Ethawi et al., 2016). In infants with persistent pulmonary hypertension, HFJV and HFOV led to similar outcomes once adjusted for differences in comparison groups (Coates, 2008). Other special considerations for neonates include meconium aspiration syndrome and pneumonia, in which HFJV may benefit from being combined with surfactant (Davis et al., 1992; Calkovska et al., 2005).

## Discussion

Although the results of this review are seemingly bleak, with no consistent outcomes among studies or determination of optimal modes or methods of setting them, a few conclusions may be drawn. The first is that caution must be taken when interpreting studies and comparing modes against one other, particularly when a protocol for setting a mode has not been established, and when comparing studies spanning large periods of time. The second is that there may not be a single optimal mechanical ventilation approach. The best method may simply be one that the intensivist is comfortable with and one that allows for an adaptive approach so the settings are personalized to the individual patient and disease pathophysiology (Gattinoni et al., 2016). Finally, when considering study design, not only does the number of enrolled centers and patients need to be considered, but also patient phenotype. A well-designed multi-center study would ideally also have a centralized method of providing continuous oversight of ventilator settings and waveforms of patients across participating institutions to standardize ventilator adjustments and maximize internal validity.

There has been a call for an increased number and quality of RCTs in pediatric and neonatal mechanical ventilation trials. With the lower incidence of lung injury in pediatric and neonatal patients, it is well-accepted that a properly powered study would require involvement of several institutions over a few year period, not only creating difficulty maintaining inter-institutional protocols and compliance but also over an extended period of time (Khemani and Newth, 2010). Future pediatric mechanical ventilation studies must be carefully designed, not only to ensure adequate power, but also with appropriate age stratification and inclusion/exclusion criteria. Even a perfectly controlled multi-institutional RCT can prompt the question of whether the average of a population with varying clinical characteristics and disease phenotypes can be applied to the individual patient (Goligher et al., 2015). The most prominent example of this is in patients with extrapulmonary vs. pulmonary lung injury. These two quite distinct phenotypes are both lumped together under the umbrella of ARDS yet patients with one vs. the other will respond disparately to ventilator setting adjustments (Kollisch-Singule et al., 2018). Not only should patients be partitioned into pediatric and neonatal categories, but also specific age groups to account for changes in chest wall stiffness and alveolarization. Patients should also be analyzed according to lung injury phenotype in pediatric patients (pulmonary vs. extrapulmonary) and disease type in neonates (persistent pulmonary hypertension, respiratory distress syndrome, congenital heart disease, meconium aspiration syndrome) and whether or not they are on ECMO.

One of the great challenges with RCTs is that a clinician may be expected to set and adjust a mechanical ventilator mode that they are not accustomed to. All clinicians are hostage to their experience, both in training and with previous patients. It is unrealistic to expect improved outcomes in a mode that a clinician is uncomfortable with, particularly if the methods are not well-protocolized. The “best” mechanical ventilation mode is not only dependent on the patient and disease pathophysiology, but also on the experience of the individual making the ventilator adjustments. Therefore, to make an RCT successful requires teaching modules, clinician humility, and consideration toward making ventilator adjustments in a coordinated team fashion.

## Conclusion

In summary, there are a variety of mechanical ventilation modes available for neonatal and pediatric use, each with benefits and drawbacks but with no definitive indications or protocols for use. In part, this is due to the lack of definitive evidence from trials, however, Froese (Froese and Kinsella, 2005) well-articulated that “*a premature trial can kill a good technique (almost)*.” More trials may not provide the answers we are searching for and may provide more conflicting data, particularly without thoughtful study design. There may never be one universalized mechanical ventilation protocol that can be safely and effectively applied to all pediatric and neonatal patients, but it is important to be open to additional strategies and understand the fundamentals of each so they may be titrated to the individual patient. We will likely find that the best mechanical ventilation strategy is that which is personalized and adaptive to the patient.

## Author Contributions

MK-S: manuscript drafting. GN, PA, JS, HR, SB, LG, NH, and AB: critical revisions. All authors contributed to the article and approved the submitted version.

## Conflict of Interest

PA, GN, MK-S, and NH have presented and received honoraria and/or travel reimbursement at event(s) sponsored by Dräger Medical Systems, Inc. outside of the published work. PA, GN, MK-S, LG, and NH have lectured for Intensive Care Online Network, Inc. (ICON). NH is the founder of ICON, of which PA is an employee. NH holds patents on a method of initiating, managing and/or weaning airway pressure release ventilation, as well as controlling a ventilator in accordance with the same, but these patents are not commercialized, licensed or royalty-producing. AB is the founder and CEO of CircuitLife™. MK-S has received a research grant from Dräger Medical Systems, Inc. The remaining authors declare that the research was conducted in the absence of any commercial or financial relationships that could be construed as a potential conflict of interest.

## Publisher’s Note

All claims expressed in this article are solely those of the authors and do not necessarily represent those of their affiliated organizations, or those of the publisher, the editors and the reviewers. Any product that may be evaluated in this article, or claim that may be made by its manufacturer, is not guaranteed or endorsed by the publisher.
